# A multi-marker test based on family data in genome-wide association study

**DOI:** 10.1186/1471-2156-8-65

**Published:** 2007-09-25

**Authors:** Zhaogong Zhang, Shuanglin Zhang, Qiuying Sha

**Affiliations:** 1 Department of Mathematical Sciences, Michigan Technological University, Houghton, MI 49931, US; 2 School of Computer Science and Technology, Heilongjiang University, Harbin, 150080, China; 3 Department of Mathematics, Heilongjiang University, Harbin, 150080, China

## Abstract

**Background:**

Complex diseases are believed to be the results of many genes and environmental factors. Hence, multi-marker methods that can use the information of markers from different genes are appropriate for mapping complex disease genes. There already have been several multi-marker methods proposed for case-control studies. In this article, we propose a multi-marker test called a Multi-marker Pedigree Disequilibrium Test (MPDT) to analyze family data from genome-wide association studies. If the parental phenotypes are available, we also propose a two-stage test in which a genomic screening test is used to select SNPs, and then the MPDT is used to test the association of the selected SNPs.

**Results:**

We use simulation studies to evaluate the performance of the MPDT and the two-stage approach. The results show that the MPDT constantly outperforms the single marker transmission/disequilibrium test (TDT) [1]. Comparing the power of the two-stage approach with that of the one-stage approach, which approach is more powerful depends on the value of the prevalence; when the prevalence is no less than 10%, the two-stage approach may be more powerful than the one-stage approach. Otherwise, the one-stage approach is more powerful.

**Conclusion:**

The proposed MPDT, is more powerful than the single marker TDT. When the parental phenotypes are available and the prevalence is no less than 10%, the proposed two-stage approach is more powerful than the one-stage approach.

## Background

Complex diseases are presumed to be the results of many genes and environmental factors, with each gene only having a small effect on the disease. To test for the association, multi-marker methods that can combine the information of markers from different genes or across the genome are appropriate. To search for a set of susceptibility genes across the whole genome that is responsible for a complex trait, we need a multi-marker test (applicable to linked and unlinked markers) and a searching algorithm.

In case-control studies, several multi-marker association tests have been proposed which include the Hotelling's T^2 ^test proposed by Xiong et al. [2] and the score test proposed by Chapman et al. [3] and Wallace et al. [4], among others. All these methods can combine information from multiple markers in one candidate gene, different genes, or across the genome. Although several haplotype-based methods have been proposed for a family-based design [5-12], those methods can only deal with the markers in a candidate gene or a tightly linked chromosome region. Xu et al. [13] first proposed a multi-marker method dealing with unlinked markers. The test statistic of Xu et al.'s method is the weighted sum of the single marker test statistics and the weights were calculated by using the information of parental phenotypes. This method is mainly developed for quantitative traits and requires parental phenotypes.

In this article, we proposed a multi-marker association test for family-based designs. Our method, proposed for qualitative traits, does not require parental phenotypes and can deal with markers from different genes or across the genome. The proposed MPDT, as an extension of the Pedigree Disequilibrium Test (PDT) [14], can be applied to any size of pedigrees. To apply the MPDT to genome-wide association studies, we also propose a searching algorithm. The proposed multi-marker association test together with the searching algorithm allows one to search for a set of susceptibility genes across the genome responsible for a complex trait. If the parental phenotypes are available, we propose a two-stage test by using the same family-based data set. In the two-stage approach, we first use a single marker test by contrasting parental cases with parental controls to screen the SNPs, and then use the MPDT and the searching algorithm to search for a set of susceptibility genes. The two-stage approach is motivated by the method recently proposed by Steen et al. [15]. In mapping quantitative trait loci using family data, Steen et al. proposed an interesting approach that performs a SNP screening and association test using the same sample. The basic idea of Steen et al.'s method is that the screening test based on the traits and between-family genotype scores is statistically independent of the association test which depends on trait values and within-family genotype scores. The screening test is used first to select SNPs, and then the association test is performed on a much smaller set of the selected SNPs. Our two-stage approach uses a similar idea but different tests in both stages.

We use simulation studies to evaluate the performance of our proposed method. The results show that the MPDT (either one-stage or two-stage) has the correct type I error rates. In all the cases that we considered, the MPDT is more powerful than the single marker TDT. Comparing the power of the two-stage approach that uses parental phenotypes with that of the one-stage approach, which approach is more powerful depends on the value of the prevalence; when the prevalence is no less than 10%, the two-stage approach may be more powerful than the one-stage approach. Otherwise, the one-stage approach is the more powerful one.

## Methods

In this section, we will first give the test statistic of the MPDT. Then, we will discuss a searching algorithm and how to find a set of susceptibility genes by the searching method and the MPDT. Finally, we will describe a two-stage approach used to incorporate the information of parental phenotypes if it is available.

### The MPDT

As the PDT proposed by Martin et al. [14], the MPDT is designed for pedigrees of any size. In the following discussion, for simplicity of presentation, we will only give the statistic for nuclear families with affected children. It is straightforward to extend the statistic for general pedigrees. Suppose we have genotyped *m *markers across the genome or in a candidate region for each sampled individual. Consider a sample of *n *nuclear families with *n*_*i *_affected children in the *i*^*th *^family. For a biallelic marker with two alleles A and a, we code the three genotypes aa, Aa, and AA as 0, 1, and 2, respectively.

Let *F*_*ij*_, *M*_*ij *_and *u*_*ijk *_denote the genotype codes of the father, mother and *k*^*th *^child in the *i*^*th *^family at the *j*^*th *^marker, respectively, *i *= 1, 2,..., *n*; *j *= 1, 2,..., *m*; *k *= 1, 2,..., *n*_*i*_. Considering each affected child as a case, we define a pseudo-control matching each case. The pseudo-control matching the *k*^*th *^child in the *i*^*th *^family has a genotype code $u_{ijk}^{c}$ at the *j*^*th *^marker where $u_{ijk}^{c}$ is the genotype code of the two alleles not transmitting to the *k*^*th *^child by the parents. For example, if the genotypes of the father, mother, and a child are Aa, Aa, and AA, respectively, then the pseudo-control matching this child has a genotype of aa and a genotype code of 0.

It is easy to see that the genotype codes of parents, children, and the pseudo-controls have the relationship

$$u_{ijk}^{c}$$

= *F*_*ij *_+ *M*_*ij *_- *u*_*ijk*_.

Let *U*_*ijk *_= *u*_*ijk *_- $u_{ijk}^{c}$ = 2 × *u*_*ijk *_- *F*_*ij *_- *M*_*ij*_. Define a multi-marker score *U*_*ik *_for the *k*^*th *^child in the *i*^*th *^family as $U_{ik}^{T}=(U_{i1k},...,U_{imk})$ = (*U*_*i*1*k*_,..., *U*_*imk*_). The multi-marker score of the *i*^*th *^family is defined as

$$U_{i}=\sum_{k=1}^{n_{i}}U_{ik}.$$

Let $U=\sum_{i=1}^{n}U_{i}$ and $V=\sum_{i=1}^{n}U_{i}U_{i}^{T}$. The statistic of the MPDT is defined as

*T*_*C *_= *U*^*T*^*V*^⊕^*U*,

where *V*^⊕ ^is the generalized inverse of *V*. Under the null hypothesis of no association between the markers and the trait, the MPDT has approximately a *χ*^2 ^distribution with degrees of freedom *k*, where *k *is the rank of *V*. If only one marker is considered, $T_{C}=(\sum_{i=1}^{n}U_{i}{)}^{2}/\sum_{i=1}^{n}U_{i}^{2}$ is the test statistic of the PDT.

### Searching algorithm and overall p-value

In this section, we consider a genome-wide association study. Suppose we have genotyped *M *markers across the genome. Our aim is to find a set of markers that jointly have significant association with the trait. We propose two searching algorithms: Conditional Search (CS) and Sequential Forward Search (SFS). In both algorithms, each of the M markers is tested by using the PDT first. Then, the markers are ordered according to their p-values of the PDT. Suppose the p-values of markers 1, 2,..., M are in ascending order. Based on the ordered markers, the two algorithms are given below:

#### CS

The CS algorithm searches marker-sets *A*_1_,..., *A*_*L*_, where marker-set *A*_*i *_consists of markers 1,..., *i *(*i *= 1,..., *L*) and *L *is a pre-specified value. We calculate the p-value of the MPDT for each set of markers and call the p-value from this step a raw p-value.

#### SFS

The SFS algorithm begins with marker-set *A*_1 _which consists of marker 1. Then, by adding one marker to the marker-set *A*_1_, we get all of the two-locus combinations with the first marker included. We test all of the two-locus combinations by the MPDT and choose the two-locus combination with the smallest p-value (also called a raw p-value) as marker-set *A*_2_. In this way, we can get a series of marker-sets *A*_1_,..., *A*_*L*_.

Both of the two searching algorithms give a series of candidate marker-sets and the corresponding raw p-values of the MPDT. The problems that remain are choosing the "best" or the final marker-set and evaluating the overall p-value of the final marker-set. An intuitive idea is to choose the marker-set with the smallest raw p-values as the final marker-set and use a permutation procedure to evaluate the overall p-value. However, our simulation studies (results not shown) show that in most cases, the more markers a marker-set contains, the smaller the p-value of the marker-set will be. Thus, instead of using the raw p-values, we propose to use a permutation procedure recently proposed by Ge et al. [16] and further discussed by Becker and Knapp [17] to adjust the raw p-values and use the adjusted p-values to choose the final marker-set. This procedure also gives the overall p-value of the final marker-set. Let *A*_1_,..., *A*_*L *_denote the candidate marker-sets and *P*_01_,..., *P*_0*L *_denote the associated raw p-values of the MPDT. The permutation procedure includes the following steps:

1. Generate *S *(say, 1,000) permuted datasets. In each permutation, there is a 50% probability of changing the multi-marker genotype (the genotype across all of the M markers) of each child with that of the corresponding pseudo-control. The reason that we changed the genotypes across M markers simultaneously is to keep the LD structure in each permuted data set.

2. For each permuted data set, search for the *L *candidate marker-sets by either of the two algorithms. Based on the permutated data set, test for the association between each marker-set and the trait using the MPDT. For the *s*^*th *^permuted data set, denote the L candidate marker-sets by *A*_*s*1_,..., *A*_*sL *_and the associated raw p-values by *P*_*s*1_,..., *P*_*sL*_. Then, the adjusted p-value corresponding to the candidate marker-set *A*_*i *_is estimated by $p_{0l}=\frac{1}{S}\sum_{s=1}^{S}I(P_{sl}<P_{0l})$, where *I*(·) is a indicator function. We will choose the marker-set with the smallest adjusted p-value, *p*_0 _= min(*p*_01_, *p*_02_,..., *p*_0*L*_), as the final marker-set.

3. To evaluate the overall p-value of the final marker-set, we first adjust the raw p-values *P*_*s*1_,..., *P*_*sL *_for the *s*^*th *^permuted data, *s *= 1,..., *S*. The adjusted value of *P*_*sl *_is given by $p_{sl}=\frac{1}{S}\sum_{t=0}^{S}I(P_{tl}<P_{sl})$. Let *p*_*s *_= min{*p*_*s*1_,..., *p*_*sL*_}. Then, the overall p-value of the final marker-set is given by

(1) $$p_{overall}=\frac{1}{S}\sum_{s=1}^{S}I(p_{s}<p_{0}).\;\left(1\right)$$

Usually, *p*_*overall *_is obtained through another layer of permutation by a standard double permutation procedure, according to Ge et al. [16], *p*_*overall *_can be estimated by (1), which needs only one layer of permutation.

### A two-stage approach to incorporate parental phenotypes

If parental phenotypes are available, we propose a two-stage approach to incorporate the parental phenotypes. The basic idea of the two-stage approach is that the test used in the first stage is independent of the association test used in the second stage; the test in the first stage is used to select promising SNPs, and the association test in the second stage can be performed on a smaller set of the selected SNPs.

#### Stage one

The test that we propose to use in this stage is based on a test statistic for a case-control study. Consider a case-control study with N_1 _cases and N_2 _controls, and each sampled individual has a genotype at a bi-allelic marker with two alleles A and a. To test the association between the marker and the disease, one can use the test statistic

$$T_{p}=\frac{{\left(p-q\right)}^{2}}{\sigma^{2}},$$

where *p *and *q *are the sample frequencies of allele A in cases and controls, respectively; $\sigma^{2}=(\frac{1}{2N_{1}}+\frac{1}{2N_{2}})p_{0}(1-p_{0})$ is the estimated variance of *p *- *q*; *p*_0 _is the sample frequency of allele A in the whole sample. Under the null hypothesis of no association, this test statistic asymptotically follows a Chi-squared distribution with one degree of freedom. To use this test statistic in the first stage, we consider the affected parents of the sampled nuclear families as cases and the unaffected parents of the sampled nuclear families as controls. We propose to use the statistic *T*_*p *_on each of the M markers and get a corresponding P-value for each marker. Select *M*_1 _markers with the smallest P-values, where *M*_1 _is a pre-specified number, which usually is smaller than M. We will discuss how to choose *M*_1 _later. In this stage, we use only the parental information of the nuclear families.

#### Stage two

Apply the searching algorithm (including the permutation procedure) to the *M*_1 _selected markers to find a final marker-set and the overall p-value of the MPDT to test the association between the final marker-set and the trait. Since all the calculations including searching and permutation procedure are applied to the data set of *M*_1 _markers, the calculation will be much faster than that of applying the method directly to the original M markers. If the parental phenotypes and genotypes have sufficient information to keep most of the disease susceptibility loci in the selected markers and delete many noise markers in the first stage, then the two-stage approach should be more powerful. Otherwise, the two-stage approach may lose power.

### Other method compared

We compared the proposed MPDT (plus the searching algorithms) with the single-marker TDT. We also compared the power of the tests using (two-stage approach) and without using (one-stage approach) parental phenotypes. For the single marker TDT, we search for a set of significance markers by controlling the False Discovery Rates (FDRs) [18], the ratio of the number of falsely rejected null hypotheses to the total number of rejected null hypotheses. We use the one-stage approach as an example to explain the procedure. Calculate the TDT for each of the M markers and denote the ordered p-values by *p*_(1)_,..., *p*_(M)_. Declare a marker significant if the P-value of the TDT at this marker is less than a threshold *δ*_*M *_such that the FDR can be controlled at a level of *α*. The threshold *δ*_*M *_is determined by $\delta_{M}=\max\{p_{\left(i\right)}:p_{\left(i\right)}\leq \frac{i\alpha }{M}\}$ The marker set that consists of all the markers associated with the trait is called the final marker-set.

## Results

We use two sets of simulation studies to evaluate the type I error rates and the power of the proposed methods. The type I error rates are evaluated for both one-stage and two-stage approaches. For the power comparisons, we compare the proposed multiple-marker approach with the single-marker approach, and the one-stage approach with the two-stage approach. In the simulation studies, we consider nuclear families with one affected child and use *L *= 15, where *L *is the maximum number of markers contained in the searched marker-sets. To identify a set of markers, we propose two searching algorithms: CS and SFS. The results for type I error rates and power comparisons by using CS and SFS are very similar. We only show the results using the CS algorithm.

In the first set of simulation studies, we use the data sets where the nearby markers are in Linkage Disequilibrium (LD). For the purpose of generating genotypes with LD between markers, we use Hudson's program (ms software) [19] which assumes the coalescent process with recombination to generate multi-marker haplotypes. We follow Nordborg M, Tavare S [20] and Kimmel G, Shamir R's [21] methods, using a mutation rate of 2.5 × 10^-8 ^per nucleotide per generation, a recombination rate of 10^-8 ^per pair of nucleotides per generation, and an effective population size of 10,000 individuals. Of all the segregating sites, only the ones with minor-allele frequency >5% are defined as SNPs and are used in the rest of the analysis. The simulation program produces populations from which samples of cases and controls can be drawn.

In the second set of simulation studies, we generate the genotypes by assuming the Hardy-Weinberg equilibrium and linkage equilibrium. This means that we generate each allele at each marker independently, and the minor allele frequency at each marker is drawn randomly between 0.05~0.5.

### Assessing the type I error rates

To assess the type I error rates, we generate data under the null hypothesis of no association between trait values and the multi-marker genotypes. In both of the two sets of simulations, we first generate the genotypes of the parents of each nuclear family and then each parent randomly transmits one of the two haplotypes to the child to form the child's genotype. We consider the sample size of 500 nuclear families and three different numbers of markers: 100, 1,000 and 100,000. For each simulation scenario, we generate 1,000 samples to estimate the type I error rates of the MPDT and TDT for one-stage approaches as well as two-stage approaches by using different values of *M*_1 _(the number of markers retained in the first stage). Note that when *M*_1 _= *M*, the two-stage method is, in fact, the one-stage approach. For 1,000 replications, the 95% confidence interval of the type I error rate is (0.0362, 0.0638) with a nominal level of 5%. The results are summarized in Table 1. Table 1 shows that the type I error rates of the MPDT and TDT are very consistent with the nominal level of 5% for both one-stage approaches (the case of *M*_1 _= *M*) and two-stage approaches (*M*_1 _<*M*). We can see that the consistency does not depend on the value of *M*_1_. The correct type I error rates for the two-stage approach also show that the tests used in the first stage and in the second stage should be independent.

**Table 1:** Type I error rates of the MPDT and TDT

M	M_1_	With LD between markers		Without LD between markers	
		
		TDT	MPDT	TDT	MPDT
100	1	0.051	0.040	0.052	0.049
	5	0.053	0.046	0.057	0.061
	10	0.052	0.051	0.045	0.049
	50	0.044	0.053	0.039	0.039
	100	0.046	0.061	0.039	0.051
1,000	5	0.055	0.061	0.048	0.042
	10	0.052	0.037	0.040	0.061
	50	0.048	0.061	0.044	0.041
	100	0.049	0.042	0.048	0.044
	1,000	0.045	0.059	0.059	0.042
100,000	1,000	0.053	0.040	0.045	0.057
	10,000	0.053	0.050	0.042	0.053
	30,000	0.039	0.045	0.047	0.050
	50,000	0.039	0.049	0.042	0.056
	100,000	0.053	0.060	0.049	0.052

*M *is the total number of markers and *M*_1 _is the number of markers retained in the first stage.

### Simulation studies for evaluating power

For the power comparisons, we compare the proposed multiple-marker approach with the single-marker approach, and the one-stage approach with the two-stage approach. First, we clarify the meaning of powers for different methods.

#### Power and Power Calculation

To estimate the power of the MPDT and TDT for one-stage approaches as well as two-stage approaches, we use 100 replicated samples in both sets of simulation studies. Suppose that there are M biallelic markers and among the M markers there are m disease loci. For the MPDT (one-stage or two-stage approaches), there is a final marker-set and an overall p-value of the test for testing the association between the final marker-set and the trait. Let *s*_*i *_and *p*_*i *_denote the number of disease loci contained in the final marker-set and the overall p-value of the test to test the association between the final marker-set and the trait, respectively, for the ith replicated sample. Then, the estimated power of the MPDT is given by $power=\frac{1}{100m}\sum_{i=1}^{100}s_{i}I_{\left\{p_{i}\leq \alpha \right\}}$, where *I*_(·) _is an indicator function and *α *is the significance level. The TDT also gives a final marker-set that contains all the markers significantly associated with the trait. Let *s*_*i *_denote the number of disease loci contained in the final marker-set of the TDT. Then, the estimated power is given by $power=\frac{1}{100m}\sum_{i=1}^{100}s_{i}$. In other words, the power of the MPDT is the percentage of disease loci contained in the final marker-sets that have significant association with the trait, and the power of the TDT is the percentage of disease loci contained in the final marker-sets.

#### Power comparisons

To assess the power of the proposed method, we generate genotype data under four different disease models. In model I and model II, we consider two three-locus disease models, and in model III and model IV, we consider two ten-locus disease models. These models are similar to those used by Millstein et al. [22] in their simulation studies. Let *p *= *P*(Affected|genotype) denote the penetrance and *x*_*k *_denote the numerical code of the genotype at the *k*^*th *^disease locus. The relationship between *p *and genotype codes at the disease loci is given by the logistic models:

$$\begin{array}{l} \log\frac{p}{1-p}=\beta_{0}+\beta_{1}x_{1}+\beta_{123}x_{1}x_{2}x_{3},\\ \log\frac{p}{1-p}=\beta_{0}+\beta_{1}x_{1}+\beta_{2}x_{2}+\beta_{3}x_{3},\\ \log\frac{p}{1-p}=\beta_{0}+\sum_{i=1}^{6}\beta_{i}x_{i}+\beta_{7}x_{7}x_{8}+\beta_{8}x_{9}x_{10},\text{ and}\\ \log\frac{p}{1-p}=\beta_{0}+\sum_{i=1}^{10}\beta_{i}x_{i} \end{array}$$

for model I, II, III, and IV, respectively. The values of coefficients (except for *β*_0_) are given in Table 2. *x*_*k *_is coded by an additive coding scheme i.e. *x*_*k *_= 0, 1, or 2 corresponding to genotype *a*_*k*_*a*_*k*_, *A*_*k*_*a*_*k*_, or *A*_*k*_*A*_*k *_at the *k*th disease locus, where *A*_*k *_is the high risk allele. The value of *β*_0 _can be determined by the values of the other parameters and population prevalence. We use three different values of population prevalence: 5%, 10%, and 20%. In our simulation studies, we assume that the frequency of the minor allele (high risk allele) at each disease locus is between 0.1 and 0.33. The four models represent different interaction structures. Model II and IV represent additive effects, while Model I and III represent additive as well as interaction effects.

**Table 2:** Parameters of the four models

	Logistic model	Values of the parameters
Model I	$\log\frac{p}{1-p}=\beta_{0}+\beta_{1}x_{1}+\beta_{123}x_{1}x_{2}x_{3}$	*β*_1 _= log(2), *β*_123 _= log(5)
Model II	$\log\frac{p}{1-p}=\beta_{0}+\beta_{1}x_{1}+\beta_{2}x_{2}+\beta_{3}x_{3}$	*β*_1 _= *β*_2 _= *β*_3 _= log(2)
Model III	$\log\frac{p}{1-p}=\beta_{0}+\sum_{i=1}^{6}\beta_{i}x_{i}+\beta_{7}x_{7}x_{8}+\beta_{8}x_{9}x_{10}$	*β*_*i *_= log(2), *i *= 1,..., 6; *β*_7 _= *β*_8 _= log(3)
Model IV	$\log\frac{p}{1-p}=\beta_{0}+\sum_{i=1}^{10}\beta_{i}x_{i}$	*β*_*i *_= log(2), *i *= 1,..., 10

We generate genotypes at 100,000 markers for each individual. To generate multi-marker genotypes of parents and the affected child in each nuclear family, we use a reject/accept procedure. Using the case of genotypes with LD as an example, for each parent, we randomly choose two haplotypes with replacement from the haplotype population generated by Hudson's *ms *program to form the multi-marker genotype of each parent. Then, each parent randomly transmits one of the two multi-marker haplotypes to the child to form the child's multi-marker genotype. Let G denote the multi-marker genotype of the child at the disease loci and let *f*_*G *_= Pr(*affected*|*G*) denote the penetrance that can be calculated from a given disease model. Then, the child is affected with a probability of *f*_*G*_. If the child is affected, we will retain the family. Otherwise, we will discard the family.

**Figure 1:**
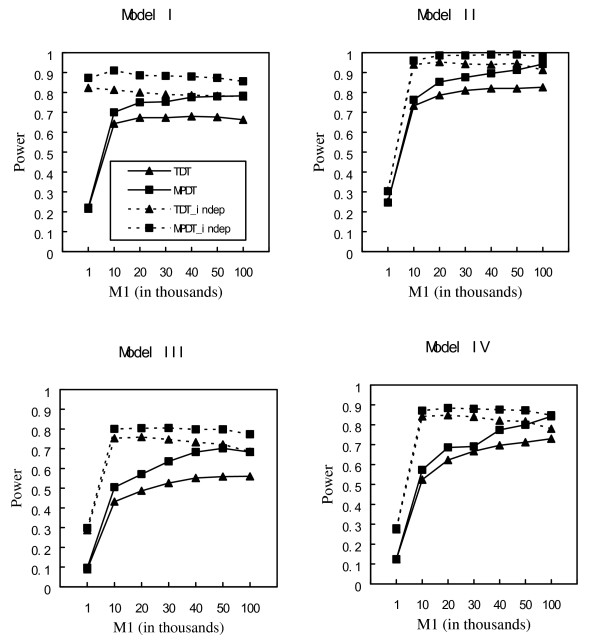
Power comparisons using the population prevalence of 0.05. TDT and MPDT denote the two tests based on the first set of simulation studies, generating genotypes with LD between markers; TDT_indep and MPDT_indep denote the two tests based on the second set of simulation studies, generating genotypes by assuming the Hardy-Weinberg equilibrium and linkage equilibrium.

**Figure 2:**
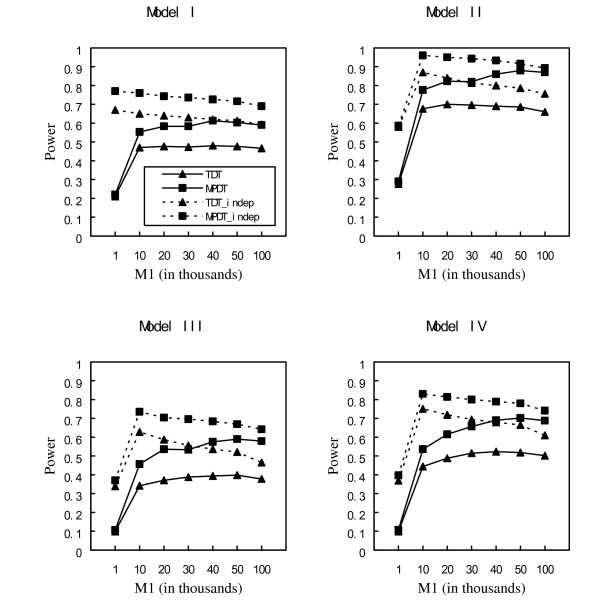
Power comparisons using the population prevalence of 0.1. TDT and MPDT denote the two tests based on the first set of simulation studies, generating genotypes with LD between markers; TDT_indep and MPDT_indep denote the two tests based on the second set of simulation studies, generating genotypes by assuming the Hardy-Weinberg equilibrium and linkage equilibrium.

**Figure 3:**
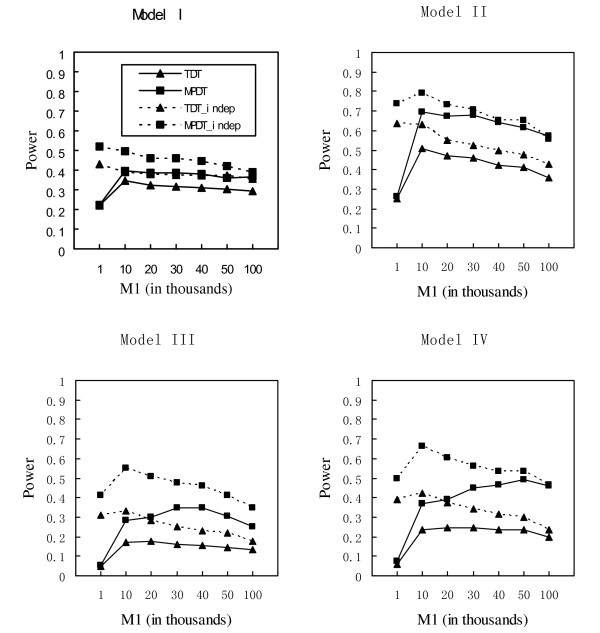
Power comparisons using the population prevalence of 0.2. TDT and MPDT denote the two tests based on the first set of simulation studies, generating genotypes with LD between markers; TDT_indep and MPDT_indep denote the two tests based on the second set of simulation studies, generating genotypes by assuming the Hardy-Weinberg equilibrium and linkage equilibrium.

In our simulations, we vary the value of *M*_1_, the number of markers retained in the first step, from 1,000 to 100,000. The results of *M*_1 _= 100,000 are the results of the one stage approaches. The power comparisons of the MPDT and TDT for one-stage and two-stage approaches with different prevalence, different LD patterns, and different models are given in Figure 1, Figure 2 and Figure 3. Comparing the MPDT with the TDT, the figures show that the multi-marker method, MPDT, is consistently more powerful than the single marker method, TDT, in all the scenarios we considered. The power comparisons of one-stage and two-stage approaches are more complicated. If there is no LD between markers, by carefully choosing *M*_1_, the two-stage approaches are more powerful than the one-stage approaches for both MPDT and TDT. However, in the more realistic situation where there is LD between markers, which approach is more powerful depends on the value of the prevalence. When the prevalence is no less than 10%, the two-stage approach may be more powerful than the one-stage approach. Otherwise, the one-stage approach is more powerful. Our conclusion of the power comparisons between the one-stage and two-stage approaches is different from that of Steen et al.'s which says that when *M*_1 _is smaller than 10, for all the cases they considered, the two-stage approach is much more powerful than the one-stage approach for a quantitative trait. The difference between our results for a qualitative trait and Steen et al.'s for a quantitative trait is not surprising. If the prevalence is small, 1% for example, the affected parents are probably only little more than 1% among all the sampled parents. Suppose we have sampled 1000 parents. The test *T*_*p *_used in the first stage is based on a case-control study with ~10 cases and ~990 controls. Since there are too few cases, the test *T*_*p *_should have limited power. When the value of the prevalence increases, the power of the test *T*_*p *_will increase, and then the two-stage approach may have some advantages over the one-stage approach.

We have also compared the Proportion of Non-Disease Loci (PNDL) contained in the final marker-set of the MPDT and TDT. Let *f*_*i *_(*t*_*i*_) denote the number of non-disease loci (total loci) contained in the final marker-set, and *p*_*i *_as the overall p-value of the test, to test the association between the final marker-set and the trait, respectively, for the ith replicated sample. Then, the estimated PNDL of the MPDT is given by

(2) $$PNDL=\frac{\sum_{i=1}^{100}f_{i}I_{\left\{p_{i}\leq \alpha \right\}}}{\sum_{i=1}^{100}t_{i}I_{\left\{p_{i}\leq \alpha \right\}}}.\;\left(2\right)$$

Let *F*_*i *_(*T*_*i*_) denote the number of non-disease loci (total loci) contained in the final marker-set of the TDT. Then, the estimated PNDL of the TDT is given by

(3) $$PNDL=\frac{\sum_{i=1}^{100}F_{i}}{\sum_{i=1}^{100}T_{i}}.\;\left(3\right)$$

When there is no LD between markers, the PNDLs of both the TDT and MPDT are very close to the nominal level 0.05 for different values of *M*_1_, prevalence and difference disease models. However, when there is LD between markers, the PNDL of the MPDT is much larger than that of the TDT. In this case, the MPDT has an average PNDL of 0.6 and the TDT has an average PNDL of 0.3. We need to note that when there is no LD between markers, the PNDL is the same as the FDR, but when there is LD between markers, the PNDL is different from the FDR. The FDR is defined as the proportion of falsely rejected loci (no LD with disease loci) in the final marker-set, but the PNDL is the proportion of non-disease loci in the final marker-set. Noting that the null hypothesis is no association with the disease (no LD with disease loci), non-disease loci in the final marker-set may have LD with the disease loci, and thus may not be the falsely rejected loci. Based on the above discussion, when there is LD between markers, although the PNDL of the MPDT is much larger than that of the TDT, we can not say that the FDR of the MPDT is larger than that of the TDT.

When there is LD between markers, we further define the FDR by changing *f*_*i *_and *F*_*i *_in equation (2) and (3) to be the number of markers that have no LD with the disease loci (the absolute value of LD measure *D*' less than 0.05) in the final marker-set. Using this definition, the FDRs of the MPDT and TDT are both near 0.05.

## Discussion

In this article, we proposed a multi-marker test, the MPDT, to analyze family-based data from genome-wide or region-wide association studies. The MPDT can combine information from multiple unrelated markers, while haplotype-based methods [5-12] can only combine information from nearby markers. Thus, the MPDT is more appropriate for searching for a set of susceptibility loci across the whole genome. By using simulation studies, we are able to demonstrate that the proposed multi-marker method, MPDT, consistently outperforms the single-marker method, TDT.

One remaining question in the MPDT is choosing the values of *L*, the maximum number of markers contained in the searched marker-sets. In our simulation studies, we use *L *= 15. If one has a prior knowledge of the number of disease loci, the value of *L *can be set to be that number. Otherwise, we suggest choosing *L *between 10 and 30. A large value of *L *increases computational burden, while a small value of *L *may limit the power.

If parental phenotypes are available, we proposed a two-stage approach. In the first stage a screening test based on parental phenotypes is used to select SNPs, and in the second stage the MPDT is performed to search for a set of susceptibility loci from the selected SNPs. Comparing a one-stage approach with the corresponding two-stage approach, the more powerful one depends on population prevalence and *M*_1_, the number of SNPs selected in the first stage. Our simulations show that only if the population prevalence is very high (≥10%), then the two-stage approach may outperform the one-stage approach. Another question needed to be pointed out is choosing the value of *M*_1_. In our simulation studies, we varied *M*_1 _from 1% to 100% of the total number of markers, M. The results show that 10% to 20% of the total numbers of markers are good choices for *M*_1_. In general, we need further investigations on choosing the optimal value of *M*_1_.

In this article, we proposed two searching algorithms: CS and SFS. Theoretically, if all the disease loci have detectable marginal effects, CS should be more powerful than SFS. If the disease loci can be divided into two groups: the first group consisting of loci with detectable marginal effects and the second consisting of loci with weak marginal effects, but having strong interaction effects with the loci in the first group, SFS should be the more powerful one. However, our simulation studies showed that the two methods have similar power in all the cases that we considered. Thus, we suggest using CS in practice because CS is much easier computationally than SFS.

## Competing interests

The author(s) declares that there are no competing interests.

## Authors' contributions

ZZ wrote the program, performed the simulation analysis and contributed to the manuscript preparation. SZ contributed to the conception of the study and to the manuscript preparation. QS contributed to the design of the study and to the manuscript preparation. All authors read and approved the final manuscript.
